# Influence of seamless nursing model of humanistic care on nursing quality and blood infection risk of neurosurgical ICU patients and its correlation with APACHE II score

**DOI:** 10.3389/fpubh.2022.944605

**Published:** 2022-09-09

**Authors:** Fuhua Liu, Xiaoting Wang

**Affiliations:** ^1^Neurosurgery Department, Zhejiang Hospital, Hangzhou, China; ^2^Intensive Care Unit Department, Zhejiang Hospital, Hangzhou, China

**Keywords:** humanistic care, seamless nursing, neurosurgical ICU, nursing quality, blood infection, APACHE II score

## Abstract

This research aims to explore the influence of seamless nursing mode of humanistic care on nursing quality and blood infection risk of ICU patients in neurosurgery, and the model of correlation with APACHE II score. 110 ICU patients are grouped into control set and study set, which are, respectively, given the previous routine nursing and the seamless management based on humanistic care to compare the two sets in the following aspects: nursing quality, blood infection rate, anxiety and depression extension before and after nursing, nursing satisfaction and APACHE II score, and to figure out the correlation between patient nursing quality score, and to compare blood infection and APACHE II score. Comparison and statistical analysis are used to disclose the influence and the correlation. The results show that there is not only a large negative correlation between nursing quality scores and APACHE II scores, but also a large negative correlation between the risk of blood infection and APACHE II score.

## Introduction

In the ICU treatment of neurosurgery, most patients are often confronted with the characteristics of disease severity and rapid variance in the disease so it is necessary to provide patients with timely and effective care to ensure their health and life. However, the number of ICU patients in neurosurgery and the heavy workload force nurses to undertake high psychological pressure, leading to a series of problems and errors in nursing, which, consequently, have side effects on the efficacy and prognosis (1). For neurosurgical ICU patients, most of them have gone through surgical treatment and may stay in the situation of severe physiological dysfunction and organ failure, not out of the danger period so they need further monitoring and treatment. However, surgery invasive operations such as trauma and tracheal intubation, which is required by the diseases themselves, increase the infection risk to some extent and have a serious impact on the prognosis and recovery. It is important to make the prevention and control of nosocomial infection in the neurosurgical ICU. And there is a need to find a safe and reliable method. It is one of the key clinical processes at present (2, 3). With the medicine development and the advancement in medical technology, the nursing model has been continuously updated and developed. Humanistic care is a human-oriented nursing model, and its application in the process of ICU nursing management can help patients increase compliance (4) and establish confidence in overcoming the disease. The seamless management is one of new models in modern nursing. The concept is based on modernization, which can largely ensure the integrity and effectiveness of nursing services (5, 6). However, the application of humanistic care combined with seamless management in neurosurgical ICU patients is rarely seen in present clinical practice, and there is no strong and effective basis for evaluating its efficacy. The impact of the seamless care model on the care quality and blood infection risk in neurosurgical ICU patients will now be explained in the following. Humanistic care in the treatment of critically ill patients can make nursing staff fully respect the patient's personality and human rights and meet the reasonable needs of patients. This nursing model can not only provide the humanistic care required for overall nursing, but also support the improvement of the professional quality of nursing staff. Therefore, humanistic care is the new era for nursing staff.

In this paper, 110 neurosurgical ICU inpatients admitted to our hospital from September 2020 to January 2021 are selected for research, and they are grouped into control set and study set according to the sequence of admission, with 55 cases in each set. Among them, there are 26 patients diagnosed with underlying diseases and 29 patients without underlying diseases; there were 30 males and 25 females in the research set, with an age range of 39~68, average age being 56.62 ± 4.75 for the male patients. In the routine set, there were 32 male cases, and 23 cases were female, the age range being 40–69 years old. The average age was 57.19 ± 4.86. There were 28 cases with underlying diseases, 27 cases without underlying diseases. For general clinical data such as disease, *P* > 0.05, there was no large difference between the sets. All patients signed the informed consent form, and this study was approved by the Medical Ethics Committee in our hospital.

This paper finds that the nursing quality scores of the study set, including theory, practice and comprehensiveness, were notoriously higher than those of the control set; the risk rate of blood infection during hospitalization in the study set was 5.45%, which was notoriously lower than that of the control set, 25.45%. After nursing, the SAS and SDS scores of the two sets decreased, and the research set was lower than the control set; after nursing, the APACHE II scores of the two sets were improved, and the improvement effect of the research set was notoriously better than that of the control set (*P* < 0.05); the seamless nursing model based on humanistic care can improve nurses' service attitude, improve doctor-patient relationship, provide patients with appropriate psychological care, correct patients' misunderstandings, create a positive attitude, eliminate negative emotions such as fear and anxiety, and make them treat the disease with optimism and increase the confidence in treatment. Additionally, this study also compared the nursing satisfaction of the two sets. The results found that the total nursing satisfaction of the research set was 89.09%, which was notoriously higher than that of the control set (63.64%) (*P* < 0.05). For neurological ICU patients, seamless nursing mode based on humanistic care can help improve the quality of care, reduce the risk of blood infection and APACHE II score, promote the recovery of patients, improve the prognosis of patients, and present clinical application potential.

The rest of this paper is organized as the follows: Section *Related work* discusses the related work, followed by the proposed methods in Section *Our proposed method*. Section *Experimental results and analysis* is the content of results in which the contrast between the main factors is conducted, and Section *Conclusion and the future work* concludes the paper with summary and future research directions.

## Related work

In recent years, with the continuous improvement of people's living standards, people's demand for medical care is increasing too, especially for the quality of patient care in ICU, which is directly related to the health of patients. The intensive care unit is an important place to provide emergency care for critically ill patients so a comprehensive, systematic, scientifically and professional nursing is required to provide a safer guarantee for the lives of patients (7). Therefore, strengthening effective nursing measures in the treatment of ICU patients is very important for the treatment and recovery of ICU patients. Seamless nursing management is a method that mainly includes the detailed understanding and management of the patient's condition, and the development of a targeted nursing plan (8). The good nursing management system ensures the smooth nursing process, thereby effectively improving the quality of nursing, reducing the incidence of patient infection, and enabling ICU patients to receive high-quality and safe nursing services during the treatment process. Humanistic care nursing is based on the principle of “human-oriented”, which can show more emotional respect and love for patients while carrying out routine nursing (9). The main reasons for the analysis are that after nutritional support based on the seamless nursing model based on humanistic care, the patient's immunity can be notoriously improved; the standardized application of antibiotics can effectively prevent superinfection and the formation of drug-resistant bacteria; strengthening environmental management and oral care can prevent respiratory infections; good disinfection of the urethra and catheter can effectively reduce the risk of infection and avoid urinary tract infections by closely monitoring the situation of the surgical incision and puncture site, and changing the bandage in time, as well as helping the patients turn over regularly, to effectively prevent skin and soft tissue infections (10, 11). Romero-Garcia et al. (12) explored the application value of seamless management model for nosocomial infection in surgical ICU, and found that providing appropriate seamless management nursing intervention can notoriously reduce the possibility of nosocomial infection in patients, which is consistent with some results of this study. For most ICU doctors and nurses, most of the time is highly stressful. In this regard, hospitals should actively take appropriate measures to reduce the pressure on nursing staff and provide better services for patients (13). The seamless nursing model focuses on scientific management, creating a seamless nursing management team so that ICU patients can create a rigorous, continuous, and integrated seamless nursing process before admission, during treatment, and after treatment (14, 15).

The application of seamless nursing management strengthens nursing management, makes nursing measures practical, improves the awareness and motivation of nursing staff, and enhances the sense of responsibility of nursing staff. At the same time, nursing responsibility can notoriously improve the quality of nursing through perfect supervision (16). It is a new nursing model that is welcomed by patients and recognized by the society (17). The application of seamless management can effectively care for the psychology of patients and improve the bad mood of patients, enable nursing staff to communicate well with patients, improve patients' confidence in nursing staff, and reduce the occurrence of medical disputes, with employees' awareness of risk prevention greatly enhanced, preventing risk events before they occur, controlling every reference and detail in nursing work, and notoriously reducing the incidence of nursing risk events while providing patients with safe, high-quality, and comprehensive nursing services (18, 19).

## Our proposed method

For the selection of patients, there are two standards including inclusion and exclusion.

The inclusion criteria include the following aspects: (1) All patients were neurosurgical ICU patients; (2) The selected patients were over 18 years old; (3) The patients had barrier-free consciousness and could communicate and communicate with doctors normally; (4) All patients signed informed consent notice.

The exclusion criteria include the following aspects: (1) Patients with poor treatment compliance and unable to determine the curative effect; (2) Patients with severe illness requiring emergency treatment; (3) Patients with severe complications; (4) Patients who have used contraindicated drugs; (5) Patients with missing data on cases and related impact examinations; (6) Patients' condition deteriorated suddenly during the research process; (7) Patients with other diseases affected the research results; (8) Patients who had unexpected circumstances during the research process, made it difficult to continue the research; (9) Serious disease during the research process affected other researchers.

### Control set

The set of patients can use the previous routine nursing mode, and the details are as follows: (1) closely monitor and observe the patient's condition and vital signs, evaluate the patient's physical responses according to the patient's actual situation, and give appropriate medication or (2) during the nursing process, closely observe and record the changes in the patient's condition, and carry out routine management and monitoring; (3) if the patient's condition improves, appropriate measures should be taken, and instruct patients to carry out relevant rehabilitation exercises, and communicate with the patient's families to guide some precautions after surgery, and provide necessary health guidance or advice to reduce the incidence of postoperative complications.

### Study set

Based on the nursing in the control set, the patients in the set were given seamless management under humanistic care. The specific operation methods are as follows:

Humanistic nursing: (1) Nursing staff need to do a good job in interviewing patients, know the patient's condition through effective communication and make the evaluation, and communicate with relevant doctors; according to the patient's operation method, condition and anesthesia method, different methods are adopted to manage patients; at the same time, formulate a set of personalized precautions for patients. Since ICU patients are relatively cumbersome and large-scale, both anesthesia and surgical procedures are more complicated. Therefore, nurses need to nurse patients carefully and cautiously. (2) Nursing staff need to adjust the temperature in the ward, preferably between 22 and 25°C, and the humidity is about 55% to ensure the comfort; do a good job in keeping the patient warm, observe the blood flow in the compressed part, and record the patient's vital signs in real time; (3)nursing staff should ensure that the drainage tube is fixed securely, the infusion channel unobstructed and the exposed parts covered; after the ward, the nursing staff make handover and communication with the nursing staff in the ward, including intraoperative responses and blood loss, etc., comfort the family members, and give them spiritual encouragement.

Seamless management: (1) Preparation stage is going to establish a seamless management team to cooperate in the entire nursing process, with the deputy chief nurse or the chief nurse as the team leader responsible for management guidance and supervision. The team consists of 6 nurses. They are responsible for completing the basic nursing operations with due diligence. At the same time, they put forward corresponding suggestions according to the problems existing in the nursing process. The goal of the seamless management team is to reduce nosocomial infections. (2) Contents: ① nutritional support management: since ICU patients are relatively weak, their immunity is low, and their defense and resistance to foreign bacterial infection is weak so the first thing to do is to strengthen nutritional support for patients, which is also the most important thing to do as one of the basic nursing cares. Nursing staff provide nutritional support to patients in the following ways of parenteral support, on-site nutrition, etc., but it should be noted that in the process of nutrient solution deployment they should be prepared and used immediately. In theory, it should not exceed 24 h to avoid the deterioration of the nutrient solution and cause unnecessary impact on the patient, and the container for placing the nutrient solution should be dry and sterile; ② abdominal infection management: generally speaking, the occurrence of abdominal infection. It is usually due to self-infection so the drug susceptibility test results should be tested for the patient in time, and appropriate sensitive antibiotics should be selected for the patient, and antibiotics should be used correctly if the patient has related symptoms;③respiratory tract infection management: intraoral care: guide the patient to take half in the sitting position, use chlorhexidine solution to rinse the patient's mouth every day to ensure the cleanliness of the oral cavity; environmental management: it is necessary to purify the ambient air regularly, mainly using a turbocharger to ensure that the temperature in the ward is maintained at 23~25°, the humidity is about 55% in order to ensure that the patient's airway will not be excessively dry; a humid heat exchanger can be used to properly humidify it; ventilator management: it is necessary to place the humidifier sterile liquid and thread. The tube should be replaced regularly, and the condensed water should be cleaned up in time to avoid reverse flow; ④ urinary tract infection management: after the patient is indwelled by the catheter, the vulva and urethral orifice should be disinfected with iodine with a concentration of 0.05%. The frequency is 3 times per day. After the patient defaces, the patient's perineum should also be cleaned, and the patient's urethral opening should be scrubbed at the same time in order to shorten the indwelling time of the patient's urinary catheter as much as possible to avoid complications related to the urinary catheter. ⑤ skin and soft tissue care: for ICU patients, there will be surgical incisions and puncture sites, and the skin at this site is relatively fragile so it needs to be disinfected daily, and at the same time, use a one-time sterile applicator and require frequent replacement, closely observe the skin around the puncture site of the patient; if there is redness, pain or fever, the tissue should be taken immediately for bacterial culture to ensure that the infection of the patient can be controlled in time. For long-term bedridden patients, nursing staff should instruct patients to turn over regularly to avoid local pressure and pressure ulcers.

### Observation of indicators

Indicator observation includes the following steps:

(1) The nursing quality of the two sets was compared, and the nursing quality survey scale prepared by the hospital was used to evaluate the nursing quality. The Cronbach's alpha value of this scale is 0.787.

(2) The blood infection rate of the two sets of patients was compared, and the blood infection rate (%) = the number of blood infections / the total number of people × 100%.

(3) The anxiety and depression of the two sets of patients before and after nursing were compared. The self-rating anxiety scale (SAS) was used to determine the degree of anxiety. The scale has a total of 20 items, and the scoring standard for each item is 1–4 points. A score >50 indicates that the patient has an anxiety tendency, and the higher the score indicates a higher level of anxiety in the patient (20). The self-rating depression scale (SDS) is used to assess the degree of depression. The scale has 20 items, and the scoring standard for each item is 1 to 4 points. A score >53 points indicates that the patient is prone to depression, and the higher score indicates that the degree of depression is more severe (21).

(4) To compare the nursing satisfaction of the two sets of patients, the nursing satisfaction survey scale prepared by the hospital was used for evaluation, including 10 items such as work attitude, nursing effect, nursing technology, etc. Each item was scored as 0–10 points <95 points are satisfied, 60- <80 points are fair, and <60 points are unsatisfactory. Satisfaction = very satisfied rate + satisfaction rate. The Cronbach's alpha value of this scale is 0.763.

(5) To compare the difference in APACHE II score between the two sets, using the APACHE II scoring system (22), the vital signs, blood routine, liver and kidney function, electrolytes, blood gas analysis and Glasgow coma score within the first 24 h of ICU admission were collected (23). The total data APS (A), age adjustment (B), chronic health adjustment (C) inspection data were obtained, and all data were entered into the APACHE II scoring scale, and finally A + B + C was obtained to obtain the APACHE II score. The patients were divided into sets according to the APACHE II total score: severe set, with scores ≤ 15 points; critically ill set, between 16 and 35 points; extremely critically ill set, >35 points.

(6) The correlation between nursing quality score, blood infection and APACHE II score of patients was compared.

### Statistical methods

In this study, all the data were organized, and a corresponding database was established for it. All databases were entered into SPSS 26.0 for data processing, and the measurement data was tested for normality, expressed as ($\bar{x}$ ± s), and between sets as *t*-test; the rate is expressed as %, and the test method is χ*2*; the correlation between nursing quality, blood infection risk and APACHE II score was analyzed by Pearson; when *P* < 0.05, the difference between the data was statistically large.

## Experimental results and analysis

### Contrast of nursing quality between the two sets

The scores of nursing quality in the study set, including theory, practices and comprehensiveness were notoriously higher than those in the control set (all *P* < 0.05), as shown in Table 1.

**Table 1:** Contrast of nursing quality between two sets ($\bar{x}$ ± s, points).

**Set**	**Theoretical nursing quality**	**Quality of practice nursing**	**Integrated nursing quality**
Control set (*n =* 55)	75.25 ± 5.39	72.52 ± 5.71	73.58 ± 5.26
Study set (*n =* 55)	95.33 ± 4.16	94.16 ± 5.27	94.89 ± 4.26
*t*	−21.827	−20.654	−23.349
*P*	<0.001	<0.001	<0.001

### Contrast of blood infection rates between the two sets of patients

The risk rate of blood infection in the study set during hospitalization was 5.45%, which was notoriously lower than that in the control set, which was 25.45% (*P* < 0.05), as shown in Table 2.

**Table 2:** Contrast of blood infection rates between the two sets of patients.

**Set**	**Risk of blood infection (%)**
Control set (*n =* 55)	14 (25.45)
Study set (*n =* 55)	3 (5.45)
χ2	8.419
*P*	0.004

### Contrast of anxiety and depression in the two sets of patients before and after nursing

**Figure 1:**
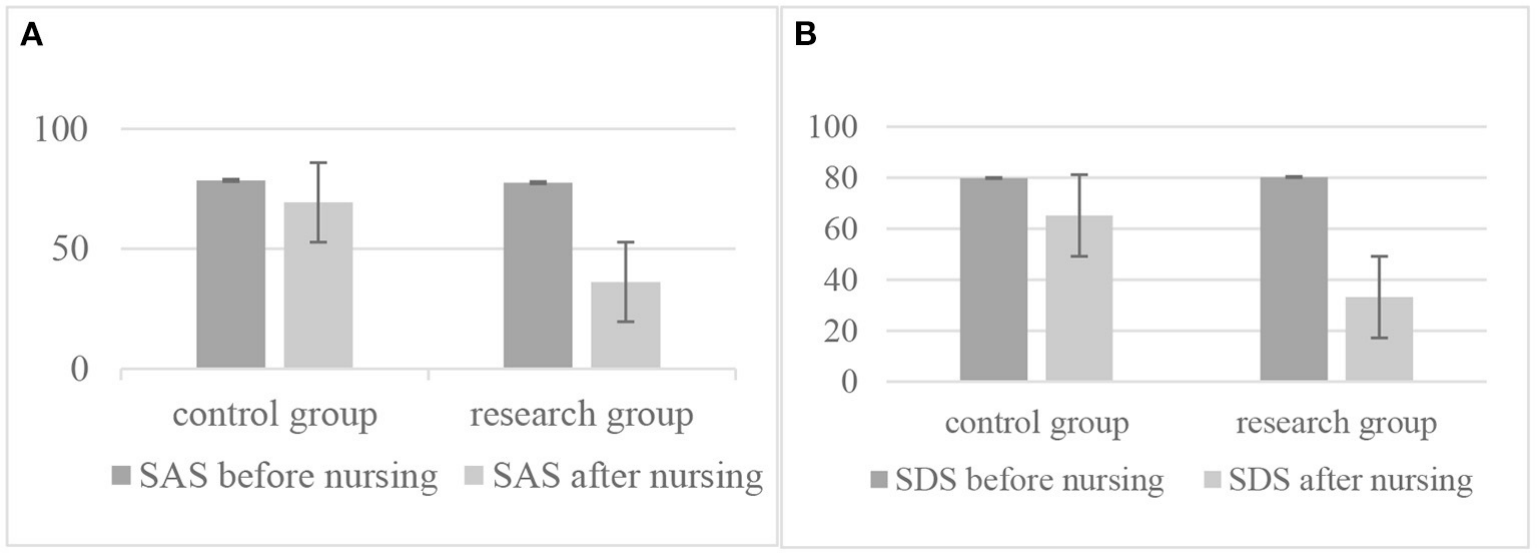
Contrast of anxiety and depression in the two sets of patients before and after nursing: **(A)** The contrast of anxiety scale between control group and research group before and/or after nursing; **(B)** The contrast of depression scale between control group and research group before and/or after nursing.

Before nursing, there was no large difference in the SAS and SDS scores between the two sets (*P* > 0.05). After nursing, the SAS and SDS scores of the two sets are decreased, and the study set is lower than the control set (*P* < 0.05), as shown in Table 3 and Figure 1.

**Table 3:** Contrast of anxiety and depression before and after nursing between the two sets of patients ($\bar{x}$ ± s, points).

**Set**	**SAS**		**SDS**	
	**Before nursing**	**After nursing**	**Before nursing**	**After nursing**
Control set (*n =* 55)	78.53 ± 4.36	69.36 ± 3.89^*^	79.85 ± 5.23	65.16 ± 4.23^*^
Study set (*n =* 55)	77.59 ± 4.45	36.23 ± 3.69^*^	80.25 ± 5.39	33.15 ± 3.19^*^
*t*	1.119	45.824	−0.395	44.808
*P*	0.266	<0.001	0.694	<0.001

Contrast with the same set before nursing,

* P < 0.05.

### Contrast of nursing satisfaction of two sets of patients

The total satisfaction with nursing in the study set was 89.09%, which was notoriously higher than that in the control set (63.64%) (*P* < 0.05), as shown in Table 4.

**Table 4:** Contrast of nursing satisfaction of two sets of patients.

**Set**	**Very satisfied**	**Satisfy**	**Generally**	**Dissatisfied**	**Satisfaction**
Control set (*n =* 55)	14 (25.45)	21 (38.18)	5 (9.09)	15 (27.27)	35 (63.64)
Study set (*n =* 55)	30 (54.55)	10 (18.18)	9 (16.36)	6 (10.91)	49 (89.09)
χ2					9.872
*P*					0.002

### Differences in APACHE II scores before and after nursing between the two sets of patients

Before nursing, the APACHE II scores of the two sets are higher, and the difference was not statistically large (*P* > 0.05), as shown in Table 5.

**Table 5:** Contrast of APACHE II scores before and after nursing between the two sets of patients ($\bar{x}$ ± s, points).

**Set**	**Before nursing**	**After nursing**
Control set (*n =* 55)	33.25 ± 5.37	22.47 ± 4.58^*^
Study set (*n =* 55)	32.93 ± 5.54	14.85 ± 2.39^*^
*t*	0.308	10.939
*P*	0.759	<0.001

Contrast with before nursing,

* means P < 0.05.

### Correlation between nursing quality score, blood infection risk and APACHE II score

#### Correlation analysis between scores of nursing quality and APACHE II score

**Figure 2:**
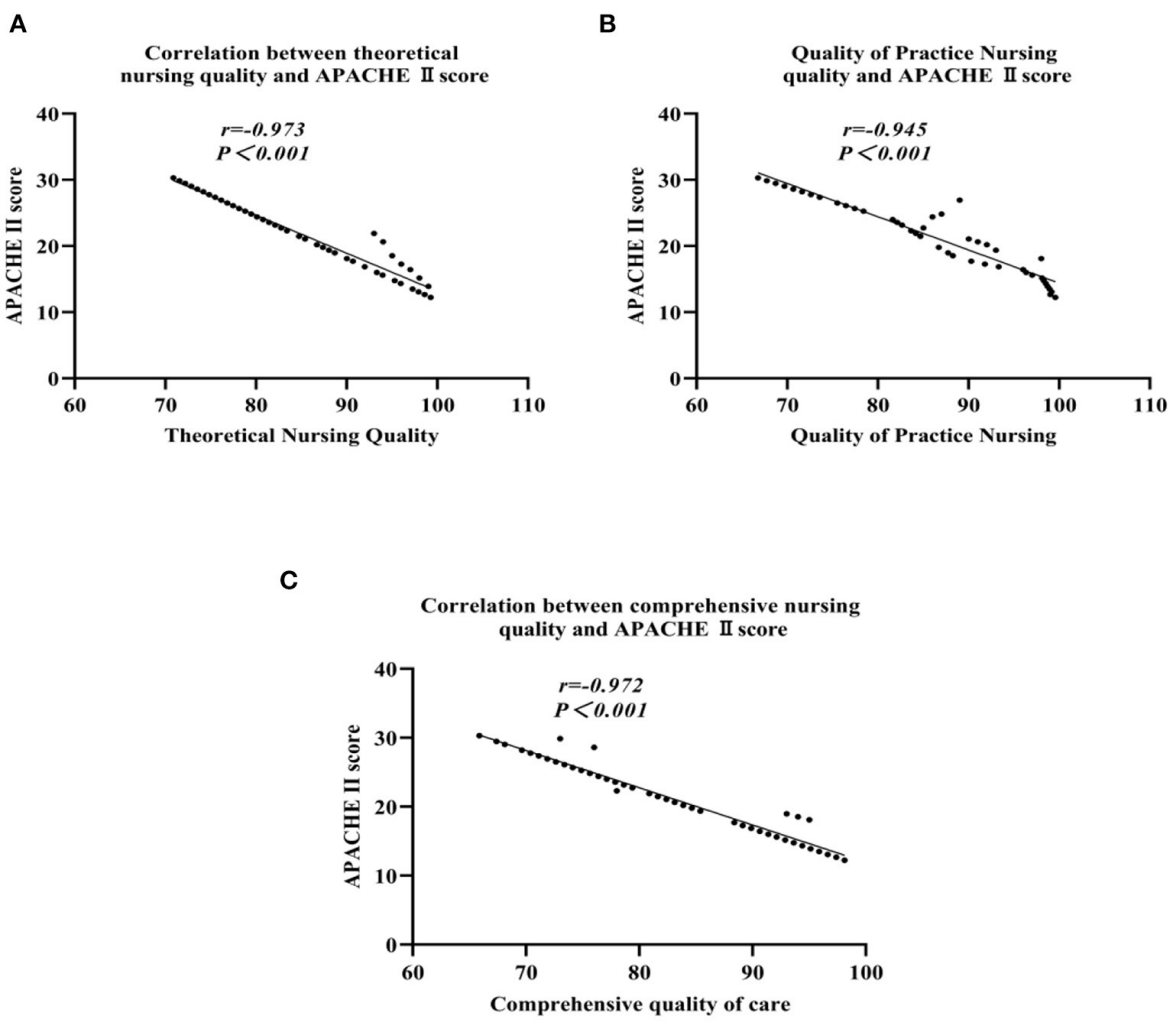
Correlation analysis of various scores of nursing quality and APACHE II score: **(A)** The theoretical nursing quality; **(B)** The practice nursing quality; **(C)** The comprehensive nursing quality.

There was a large negative correlation between various scores of nursing quality and APACHE II score (*P* < 0.05), as shown in Table 6 and Figure 2.

**Table 6:** Correlation between various scores of nursing quality and APACHE II score.

**Care quality**	***r***	***P***
Theoretical nursing quality	−0.973	<0.001
Quality of practice nursing	−0.945	<0.001
Comprehensive quality of care	−0.972	<0.001

#### Correlation between blood infection risk and APACHE II score

There was a large negative correlation between the risk of blood infection and the APACHE II score (*P* < 0.05), as shown in Table 7.

**Table 7:** Correlation between blood infection and APACHE II score.

**Index**	***r***	***P***
Risk of blood infection	−0.652	<0.001

## Conclusion and the future work

For neurological ICU patients, the seamless nursing model of humanistic care can improve their nursing quality reduce the risk of blood infection and APACHE II score, promote patient recovery, and improve patient prognosis, which is worthy of clinical application. Although this paper has achieved certain results, it still has certain limitations. Since this study did not evaluate the prognosis of patients, it is impossible to determine the long-term effect and compliance of nursing care. Therefore, in future research, the scope of sample selection should be expanded and extend the follow-up stage and further evaluate the long-term effect of the seamless nursing model of humanistic care on neurosurgical ICU patients.

## Data availability statement

The original contributions presented in the study are included in the article/supplementary material, further inquiries can be directed to the corresponding author/s.

## Ethics statement

The studies involving human participants were reviewed and approved by Zhejiang Hospital. The patients/participants provided their written informed consent to participate in this study. Written informed consent was obtained from the individual(s) for the publication of any potentially identifiable images or data included in this article.

## Author contributions

All authors listed have made a substantial, direct, and intellectual contribution to the work and approved it for publication.

## Conflict of interest

The authors declare that the research was conducted in the absence of any commercial or financial relationships that could be construed as a potential conflict of interest.

## Publisher's note

All claims expressed in this article are solely those of the authors and do not necessarily represent those of their affiliated organizations, or those of the publisher, the editors and the reviewers. Any product that may be evaluated in this article, or claim that may be made by its manufacturer, is not guaranteed or endorsed by the publisher.
